# Cannabinoid Hyperemesis Syndrome: A Rare Cause of Severe Abdominal Pain and Hyperlactataemia

**DOI:** 10.7759/cureus.92444

**Published:** 2025-09-16

**Authors:** Ahmad Elbaghdady, Abdelnasser Ahmed, Ahmed Elmahdy

**Affiliations:** 1 Acute Medicine, King's Mill Hospital/Sherwood Forest Hospitals NHS Foundation Trust, Sutton-in-Ashfield, GBR; 2 Internal Medicine, King's Mill Hospital/Sherwood Forest Hospitals NHS Foundation Trust, Sutton-in-Ashfield, GBR

**Keywords:** abdominal pain, cannabinoid hyperemesis syndrome, cannabis, capsaicin, case report, cyclic vomiting, haloperidol, hot bathing, lactic acidosis

## Abstract

Cannabinoid hyperemesis syndrome (CHS) is an emerging disorder in chronic cannabis users, characterized by recurrent nausea, vomiting, severe abdominal pain, and compulsive hot bathing. We report a case of CHS complicated by profound lactic acidosis (lactate 6.1 mmol/L), highlighting this metabolic derangement as a secondary effect of severe dehydration and physiological stress from intractable vomiting and pain. Our case underscores the importance of early recognition of CHS even when abdominal pain predominates, aggressive fluid resuscitation, and targeted therapies including haloperidol and capsaicin. We also review current evidence on CHS pathophysiology, diagnostic criteria, and management strategies, alongside the increasing prevalence in correlation with rising cannabis use in the UK.

## Introduction

Cannabinoid hyperemesis syndrome (CHS) is a paradoxical condition associated with chronic cannabis use, characterised by episodic nausea, vomiting, and severe abdominal pain often centred in the epigastrium [1,2]. First described in 2004, CHS typically presents after years of sustained cannabis exposure and is notably relieved by hot showers, a behavioural supportive feature of CHS [1,3]. Although cannabinoids exert antiemetic effects at low doses, chronic high‑dose exposure may paradoxically induce hyperemesis and intense pain via CB1 receptor overstimulation and TRPV1 activation, affecting gastrointestinal motility and pain pathways [2,4].

According to Rome IV criteria, CHS is defined by recurrent vomiting in a patient with prolonged, excessive cannabis use and resolution of symptoms upon cessation [5]. Although the Rome IV phenotype focuses on stereotypical episodic vomiting, abdominal pain is also very common in CHS (85%) and may be clinically prominent; differentiation from mimics such as cyclic vomiting syndrome, gastritis, and pancreatitis should be guided by the cannabis use context and appropriate laboratory/imaging evaluation [6]. With cannabis use on the rise, with 7.6% of UK adults aged 16-59 reporting use in 2023 [7], the burden of CHS is anticipated to grow. Increased clinician awareness is crucial for reducing morbidity, avoiding unnecessary investigations, and implementing timely management.

## Case presentation

A 32-year-old man with a 15-year history of daily cannabis smoking presented with a three-day history of severe, constant epigastric pain and intractable vomiting. He described the pain as 9 out of 10 in intensity, which was unrelieved by over-the-counter analgesics and only temporarily eased by multiple hot showers each day. He denied any use of alcohol or other drugs.

He experienced recurrent similar episodes for 15 years, requiring eight hospitalisations. He had undergone an extensive work-up with no specific positive findings, including multiple gastroscopies and a colonoscopy.

On examination, he appeared extremely restless, diaphoretic, flushed, with a heart rate of 110 beats per minute, a blood pressure of 100/60 mmHg, and a temperature of 37 °C. An abdominal examination revealed mild epigastric tenderness without any peritoneal signs.

Initial laboratory investigations revealed raised inflammatory markers and metabolic derangement. Key findings are detailed in Table 1. Notably, he had a marked neutrophilic leukocytosis, an acute kidney injury (AKI) with elevated urea and creatinine, and marked hyperlactataemia with a serum lactate level of 6.1 mmol/L. His serum amylase, liver function tests and glucose were within the normal range.

**Table 1:** Patient's laboratory investigations on admission pCO_2_: partial pressure of carbon dioxide; eGFR: estimated glomerular filtration rate

Parameter	Patient value	Reference range
Venous pH	7.540	7.35 - 7.45
Venous pCO_2_ (kPa)	3.9	5.2 - 7
Bicarbonate (mmol/L)	24.8	19 - 28
Anion gap (mmol/L)	16.0	10 - 20
Lactate (mmol/L)	6.1	0.5 - 2.5
Urea (mmol/L)	10.4	2.5 - 7.8
Creatinine (µmol/L)	153	70 - 120
eGFR (mL/min/1.73m²)	51	>90
White cell count (10^9/L)	18.4	4.0 - 10.0
Neutrophils (10^9/L)	14.7	2.0 - 7.0
C-reactive protein (mg/L)	9	<5

A contrast CT of the abdomen returned unremarkable results. Given his chronic cannabis use, cyclic vomiting, predominant severe abdominal pain, relief from hot bathing, and the exclusion of other causes, CHS was diagnosed according to the Rome IV criteria [5].

A detailed medical history was revisited, revealing that he had over 15 years of smoking cannabis, as well as vaping and minimal alcohol consumption. Notably, he described that his symptoms improved with hot showers, requiring multiple showers each day, and symptoms resolved after stopping cannabis for the past year; however, he had restarted heavy smoking of cannabis for the last two months.

Given the relationship between his symptoms and heavy cannabis use, along with supportive hot showering behaviour, a diagnosis of CHS was made.

Differential diagnosis

Alternative causes of acute abdominal pain and vomiting were systematically evaluated. Acute pancreatitis was unlikely: contrast-enhanced abdominal CT was unremarkable, and serum amylase was within the reference range. Biliary colic/obstruction was not supported by examination (no right upper quadrant tenderness) or biochemistry (no cholestatic liver enzyme pattern), and CT showed no biliary dilatation or radiologic features of cholecystitis. Bowel obstruction was not suggested clinically (no obstipation, no peritoneal signs), and CT demonstrated no obstructive transition point. Gastritis/peptic ulcer disease was unlikely given the absence of gastrointestinal bleeding and a recent normal oesophagogastroduodenoscopy. Finally, the presentation favoured CHS over cyclic vomiting syndrome (CVS): CHS occurs in the context of chronic cannabis exposure and is often accompanied by compulsive hot-water bathing with improvement on abstinence, whereas CVS lacks cannabis exposure and typically features less prominent abdominal pain.

Treatment

Initial management involved aggressive intravenous isotonic fluid administration and electrolyte repletion. Standard antiemetics, such as ondansetron and metoclopramide, were ineffective. Over 48 hours, vomiting ceased, pain significantly improved, and lactate levels, renal function, and inflammatory markers returned to normal. He was discharged with addiction counselling and was advised to stop cannabis use permanently.

## Discussion

CHS should be considered in chronic cannabis users presenting with cyclical vomiting and severe abdominal pain, particularly when hot bathing provides relief [1,3,5]. While Rome IV emphasises vomiting, recent series report that abdominal pain is present in up to 85% of CHS cases and can predominate over nausea [6]. In contrast, cyclic vomiting syndrome is idiopathic and often spares severe pain between episodes, gastritis exhibits mucosal inflammation on endoscopy, and pancreatitis shows elevated enzymes, findings absent in CHS [6].

Hyperlactataemia in CHS is most consistent with Type A lactic acidosis driven by severe volume depletion from protracted emesis and pain-related catecholaminergic surge. In our case, the elevated lactate (6.1 mmol/L) occurred with tachycardia (110 bpm), relative hypotension (100/60 mmHg), and prerenal acute kidney injury (urea 10.4 mmol/L; creatinine 153 µmol/L) and normalised rapidly with intravenous fluids-features typical of Type A lactic acidosis [2,4,8]. Similar cases report lactate levels >5 mmol/L resolving with fluids [8]. Measuring lactate can gauge severity and guide resuscitation intensity, but it is not diagnostic of CHS itself.

Pathophysiology involves chronic CB1 receptor overstimulation, slowing gastric emptying and altering hypothalamic thermoregulation, explaining the hyperemesis, pain, and hot bathing behaviour [2,4]. Genetic predisposition may modulate susceptibility, though data remain limited.

Management centres on supportive care and symptom control, aggressive IV fluids correct dehydration, renal impairment, and lactic acidosis [2,8]. Standard antiemetics often fail; dopamine antagonists such as haloperidol have demonstrated superiority in randomised trials, reducing both emesis and pain [3,9]. Topical capsaicin cream provides rapid analgesic and antiemetic effects via TRPV1 receptor activation [1,9]. Benzodiazepines may further alleviate symptoms through anxiolysis.

The only definitive treatment is sustained cannabis cessation, leading to near‑complete symptom resolution in 97% of patients [2,5]. Relapse is common if cannabis use resumes, perpetuating the cycle of vomiting and pain. Patient education and addiction support are, therefore, critical.

With 7.6% of UK adults using cannabis annually [7], CHS prevalence and related emergency visits are expected to rise. Clinicians should maintain a high index of suspicion for CHS in young adults with unexplained vomiting and severe abdominal pain, to prevent unnecessary investigations and optimise management.

**Table 2:** Diagnostic criteria for CHS AGA: American Gastroenterological Association; CVS: cyclic vomiting syndrome; CHS: cannabinoid hyperemesis syndrome

Framework	Core diagnostic elements
AGA Clinical Practice Update [10]	1. Clinical features: stereotypical episodic vomiting resembling CVS in terms of onset, with frequency 3 or more times annually; 2. Cannabis use patterns: duration of cannabis use more than 1 year before symptom onset; frequency more than 4 times per week, on average; 3. Cannabis cessation: resolution of symptoms after a period of abstinence from cannabis use for at least 6 months, or at least equal to the total duration of 3 typical vomiting cycles in that patient.
Rome IV criteria [5]	Must include all of the following: 1. Stereotypical episodic vomiting resembling CVS in terms of onset, duration, and frequency 2. Presentation after prolonged excessive cannabis use 3. Relief of vomiting episodes by sustained cessation of cannabis use criteria fulfilled for the last 3 months with symptom onset at least 6 months before diagnosis.

## Conclusions

Cannabinoid hyperemesis syndrome (CHS) is an increasingly common yet frequently underdiagnosed cause of recurrent vomiting and severe abdominal pain in individuals with a history of chronic cannabis use. CHS can present with profound abdominal pain and severe hyperlactataemia in addition to cyclic vomiting. Recognition hinges on clinical history, including chronic cannabis use and hot bathing behaviour, and exclusion of alternative diagnoses. A thorough history, including cannabis use and symptom chronology, is crucial for diagnosis. Management requires aggressive fluid resuscitation, effective antiemetics such as haloperidol, topical capsaicin for analgesia, and, critically, cannabis cessation. Early recognition and cessation of cannabis use can prevent recurrent hospitalisations and improve patient outcomes. The expense and morbidity of extensive diagnostic workups could be reduced by increased awareness of CHS.
